# A global perspective of correlation between maternal blood lead levels and risks of preeclampsia: An updated systematic review and meta-analysis

**DOI:** 10.3389/fpubh.2022.1072052

**Published:** 2022-12-23

**Authors:** Zixing Zhong, Qingmei Yang, Chu Li, Xiaohong Chen, Feifei Zhou

**Affiliations:** ^1^Center for Reproductive Medicine, Department of Obstetrics, Zhejiang Provincial People's Hospital (Affiliated People's Hospital, Hangzhou Medical College), Hangzhou, China; ^2^Graduate School, Bengbu Medical University, Bengbu, Anhui, China; ^3^Center for Reproductive Medicine, Department of Reproductive Endocrinology, Zhejiang Provincial People's Hospital (Affiliated People's Hospital, Hangzhou Medical College), Hangzhou, Zhejiang, China; ^4^The Second Clinical College of Zhejiang Chinese Medical University, Hangzhou, China; ^5^Department of Pediatrics, Central Hospital of Haining, Haining, Zhejiang, China; ^6^Center for Reproductive Medicine, Department of Traditional Chinese Medicine, Zhejiang Provincial People's Hospital (Affiliated People's Hospital, Hangzhou Medical College), Hangzhou, China

**Keywords:** lead, Pb, heavy metals, hypertensive disorder complicating pregnancy, preeclampsia (PE), systematic review

## Abstract

**Background:**

Preeclampsia (PE) is a specific hypertensive disorder in pregnancy. Lead (Pb) is a heavy metal that affects women's reproductive health. However, it is unclear whether lead exposure during can predispose maternal risk of developing preeclampsia. This systematic review and meta-analysis study aimed to explore the association.

**Methods:**

We searched studies from three databases (PubMed, Web of Science, Embase). Only case-control, cross-sectional, and cohort studies reporting maternal blood lead levels (BLL) and PE were included from database inception to 31st July 2022. Pregnant women with blood lead levels measured were eligible. Those healthy pregnant women who did not develop preeclampsia were assessed as comparators. Letters, comments, case reports, and reviews were excluded. Newcastle-Ottawa Scale (NOS) and its adaptive form were applied for assessment. The random-effects method (REM) was applied to calculate the standardized mean difference (SMD) with a 95% confidence interval (CI). Stata 16.0 and RevMan 5.3 were the software used for data extraction and analysis.

**Results:**

25 studies out of 1,808 articles made the finalist for systematic reviews, of which 21 underwent further quantity analysis. A total of 1,533 preeclamptic women and 10,998 healthy pregnant controls were included in the meta-analysis. The overall result revealed that maternal lead exposure was significantly higher in women with preeclampsia (SMD: 1.06, 95% CI 0.69, 1.43); (*I*^2^ = 96.40%; *P* = 0.000).

**Conclusion:**

This study demonstrates that maternal lead exposure is associated with preeclampsia during pregnancy. The association is present even in low blood lead levels. The conclusion should be taken seriously and women should avoid unexpected exposure to a lead-containing environment as much as possible.

**Systematic review registration:**

https://www.crd.york.ac.uk/prospero/display_record.php?RecordID=347220, identifier: CRD42022347220.

## 1. Introduction

Preeclampsia (PE) is a pregnancy-specific disorder that can affect multi-systems. It features the late-onset hypertension, proteinuria, deranged liver enzymes, blurred vision, headache, etc. Globally, the incidence of this hypertensive disorder complicating pregnancy is around 5% (1–3). It remains one of the leading causes of maternal death in most countries, particularly in developing countries. Despite progress made in early screening and prevention in PE, the management is mainly unchanged. The most effective approach to stop the disease progression is still the termination of pregnancy. This may lead to iatrogenic preterm deliveries, causing heavy economic burden for the family and the society. Moreover, as the etiology of PE remains poorly understood, some researchers proposed that heavy metals may play a role (4–9).

Lead (Pb) is one of the most toxic heavy metals in the environment (10, 11). Environmental lead exposure can be inadvertent as it is contained in batteries, cosmetics, paints, metallic pipes, and some cooking pots (10). It can affect the biological function of major organs and systems, such as the central nervous and cardiovascular systems (12). Several studies have reported an association between occupational and environmental lead exposure and hypertension (12). Exposure to lead could affect the central nervous system, causing biological functioning of enzymes, behavioral disorders and brain damage (13). The association between lead exposure and reproductive health has also been studied across countries in recent decades (14–17). Male workers exposed to lead manifest higher blood lead levels (BLL), lower sperm count, and poor sperm motility compared to those without occupational lead exposure (18). By contrast, lead-exposed women are at higher risk of developing PCOS (19).

Many studies have reported an association between heavy metals and preeclampsia during pregnancy, but the results are inconsistent (16, 20–22). Several reasons, such as different ethnic backgrounds, geographical locations, and measurement methods, may explain the disparity. Therefore, we conducted this systematic review and meta-analysis to include all eligible studies to discuss: (1) whether there is an association between maternal lead exposure and preeclampsia; (2) how maternal lead exposure may affect the risk of PE in pregnancy.

## 2. Methods

### 2.1. Protocol and registration

This study followed the Preferred Reporting Item for Systematic Reviews and Meta-analysis (PRISMA) Statement. We registered at the National Institution for Health Research with the registration identifier: CRD42022347220. https://www.crd.york.ac.uk/prospero/display_record.php?RecordID=347220.

### 2.2. Search strategy

We searched three electronic databases, PubMed, Web of Science, and Embase, from the inception to 31st Jul 2022. Two independent researchers (Z. X. Z. and Q. M. Y.) used a combination of Medical Subject Headings (MeSH) terms and free text words, e.g., “preeclampsia or pre-eclampsia or (hypertensive disorder complicating pregnancy) or (hypertensive disorder during pregnancy) or (Pregnancy-Induced Hypertension) or (gestational hypertension)” and “lead or Pb.” We have manually checked all included studies and references to complement our study. Studies were limited to humans, but there were no restrictions on language or places of study. The detailed search strategies can be accessed in Supplementary material.

### 2.3. Eligibility criteria and the study selection process

Two researchers (Z. X. Z. and Q. M. Y.) independently included studies that were: Observational studies that measure maternal blood lead levels in preeclamptic women and healthy pregnant controls.

Studies were excluded if they were conference papers, editorials, letters, reviews or systematic reviews. Z. X. Z. and Q. M. Y. screened the studies, and a third reviewer, F. F. Z., was to resolve any disagreement between the two.

### 2.4. Data extraction and quality assessment

Two independent investigators (Z. X. Z. and Q. M. Y.) extracted data *via* Microsoft Excel 2018. The title of studies, name of the authors, year of publication, study types, country of study, number of participants, blood lead levels (BLL), and average maternal age with standard deviation (SD) in each group were extracted from each study. F. F. Z. was to resolve any disagreement during data extraction between the two.

The Newcastle-Ottawa Scale (NOS) was adopted to evaluate case-control, cohort, and cross-sectional studies. A nine-star rating system was applied for quality assessment in case-control and cohort studies. A score between seven and nine indicates good quality, while four to six was considered moderate quality. Poor quality was defined if the score was three or less. For cross-sectional studies, a modified form of NOS was used. The scores rated from zero to ten. Scores of seven or more represent good quality, while three or fewer represent poor quality. Fair quality was defined as scores in between (23).

### 2.5. Sub-group analysis and meta-regression

Sub-group analysis and meta-regression were conducted to assess whether geographical locations, study or sample types, or measurement methods affected maternal exposure to lead, and how this correlated with the likelihood of preeclampsia. We divided all studies into five groups according to the original locations of the study population: Asian studies were from China and India. African studies consisted of reports from DR Congo, Egypt, and Nigeria. Middle-East studies cover reports from Iran, Saudi Arabia, and Turkey. European studies contain reports from Bulgaria, Finland, France, Malta, Poland, Portugal, and the UK. Other studies from the USA and Australia were allocated to Group 5 (Others).

We also applied meta-regression to determine whether the geographical locations, the study types, the methods of measurement, or the blood samples (the whole blood, plasma, serum) were the contributing factors to the overall results and heterogeneity.

### 2.6. Statistical analysis

We calculated results and performed data analysis *via* Review Manager 5.4.1 (The Nordic Cochrane Center, Copenhagen, Denmark) and Stata version 16.0 (StataCorp., College Station, TX, USA). The maternal blood lead exposure levels were pooled by standardized mean difference (SMD) with 95% confidence intervals (CI). The *I*^2^ was used to test the heterogeneity (*I*^2^ ≥ 50% indicates significant heterogeneity). The forest plot was used to visualize the overall results, with the random-effect model (REM) being adopted for calculation as the heterogeneity was considered significant. A sensitivity analysis was performed with the removal of each study once to assess whether any single study could affect the overall outcome. Publication bias was visualized *via* funnel plot with Begg's test and tested with Egger's linear regression.

## 3. Results

### 3.1. Study selection

**Figure 1:**
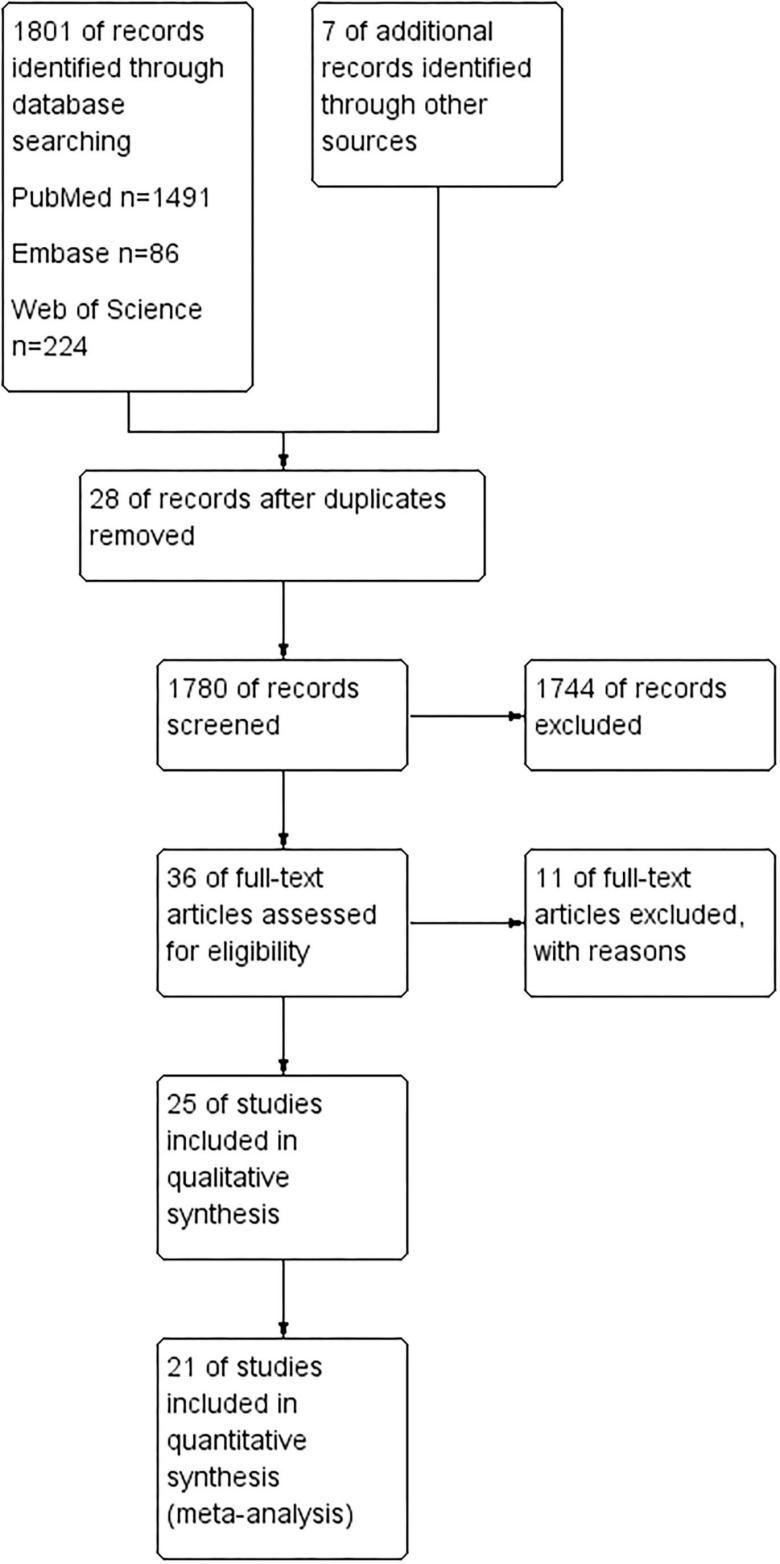
PRISMA flow diagram for study selection process.

We searched three databases (PubMed, Web of Science, and Embase) and collected 1,801 articles. Seven additional studies were identified after checking all the references from full-text articles. Of the 1,808 studies, 28 were removed for duplication. One thousand seven hundred and eighty records were further screened, and 1,744 were removed after reading their title and abstract. There were 36 studies investigated for full-text assessment. Eleven of them were excluded for reasons: Letonoff et al. study was included in a recent meta-analysis, but we excluded it from our finalist as we had checked the measurement in this study and failed to identify the type of measurement used in the article and references (24). Moreover, the diagnostic criteria were significantly changed over the eight decades (24, 25). Three studies were excluded for not reporting blood samples of lead (6, 26, 27). Three articles were excluded as they overlapped the study population with the finalist articles (28–30). The other four articles failed to make it into the finalist as they studied the association between lead exposure and obstetric outcomes during pregnancy but did not involve preeclampsia (31–34). Of the rest 25 articles, 21 were included in systematic review and meta-analysis, while four studies were only assessed in the qualitative synthesis (14–17, 20–22, 35–48). Details of the study selection process can be seen in Figure 1.

### 3.2. Basic characteristics of included studies

There are 25 studies originated from 17 countries over four decades. There are contrasting differences between the size of the study population with the number of preeclamptic participants in any single study ranging from six to 427 (14, 47). Significant differences were also found in blood samples (whole blood, plasma, serum or red blood cells), and methods of measurement, such as atomic absorption spectrophotometer (AAS), inductively coupled plasma mass spectrometry (ICP-MS) or inductively coupled plasma optical emission spectrometry (ICP-OES), etc. (37, 38, 40). Most studies adopted ACOG's diagnostic criteria of preeclampsia for patient inclusion. More information can be seen in Table 1.

**Table 1:** Characteristics of included studies.

**Reference**	**Country**	**Study**	**Age of**		**Age of**		**BLL in**	**BLL in**	**Unit**	**Measurement**	**Diagnostic**
		**type**	**preeclampsia**		**healthy control**		**preeclampsia**	**healthy control**			**criteria**
			**(Mean ±SD)**	***N***	**(Mean ±SD)**	***N***	**(Mean ±SD)**	**(Mean ±SD)**			
Bayat et al. (51)	Iran	CC	29.67 ± 6.37	NS	27.37 ± 6.1	NS	8.04 ± 3.40	6.24 ± 1.74	ug/dL	Potentiometric	ACOG
Dawson et al. (35)	USA	Cohort	22.00 ± 4.00	19	25.00 ± 6.00	20	1.73 ± 0.32	1.35 ± 0.27	umol/L	AAS	NS
Disha et al. (36)	India	CS	27.34 ± 6.40	44	24.54 ± 3.60	23	3.42 ± 2.18	2.38 ± 2.43	ug/dL	AAS	ACOG
Gajewska et al. (22)	Poland-Portugal	CC	26.80 ± 3.29	66	32.70 ± 6.18	40	3.36 ± 1.23	2.04 ± 1.30	ug/dL	ICP-MS	ACOG
Hyvonen-Dabek et al. (14)	Finland	Cohort	NS	6	NS	21	0.014 ± 0.004	0.014 ± 0.0058	ppm	PIXE	NS
Ikechukwu et al. (37)	Nigeria	Cohort	27.30 ± 3.20	59	26.70 ± 3.60	150	60.2 ± 12.8	26.30 ± 8.00	ug/dL	AAS	ACOG
Jameil et al. (38)	KSA	CC	31.55 ± 6.14	40	31.20 ± 5.84	40	27.18 ± 2.13	18.23 ± 2.34	ug/dL	ICP-OES	ACOG
Kaul et al. (39)	India	CS	NS	16	NS	84	18.40 ± 1.40	6.20 ± 2.00	ug/dL	AAS	ACOG
Liu et al. (40)	USA	PC	29.12 ± 6.17	115	27.99 ± 6.31	1,159	2.53 ± 1.20	2.61 ± 1.48	ug/dL	ICP-MS	ACOG
Ma et al. (20)	China	NCC	NS	146	NS	292	8.32 ± 3.73	7.25 ± 3.81	ug/L	ICP-MS	NICE
Magri et al. (15)	Malta	CS	30.00 ± 6.00	30	27.00 ± 6.00	93	9.60 ± 6.00	5.80 ± 3.00	ug/dL	AAS	Mounier-Vehier et al. (67)
McKeating et al. (21)	Australia	NC	31.55 ± 3.80	38	32.24 ± 3.91	193	0.29 ± 0.44	0.44 ± 1.86	ug/L	ICP-MS	Kaitu'u-Lino et al. (68)
Mokhlesi et al. (41)	Iran	Cohort	NS	20	NS	1,013	7.87 ± 4.61	4.63 ± 4.80	ug/dL	NS	ACOG
Motawei et al. (42)	Egypt	CS	NS	115	NS	25	37.68 ± 9.17	14.5 ± 3.18	ug/dL	AAS	ACOG
Obadia et al. (43)	DR Congo	CC	30.60 ± 6.40	40	31.40 ± 4.70	39	6.58 ± 2.14	5.23 ± 1.56	ug/dL	ICP-MS	ACOG
Ovayolu et al. (44)	Turkey	CC	30.61 ± 7.74	46	28.00 ± 6.59	46	39.27 ± 33.67	28.48 ± 13.06	ug/L	ICP-MS	ACOG
Rothenberg et al. (63)	USA	PC	NS	NS	NS	NS	NS	NS	ug/dL	AAS	NS
Sowers et al. (64)	USA	PC	NS	NS	NS	NS	NS	NS	ug/dL	AAS	NS
Tabacova et al. (45)	Bulgaria	Cohort	24.60 ± 6.10	19	22.70 ± 5.16	22	6.50 ± 2.18	5.20 ± 0.94	ug/dL	AAS	NS
Taylor et al. (16)	UK	PC	NS	91	NS	3976	3.63 ± 1.22	3.67 ± 1.47	ug/dL	ICP-MS	ACOG
Ugwuja et al. (65)	Nigeria	PC	NS	NS	NS	NS	NS	NS	ug/dL	AAS	ACOG
Vigeh et al. (46)	Iran	CC	26.00 ± 4.00	31	26.90 ± 5.70	365	5.09 ± 2.01	4.82 ± 2.22	ug/dL	ICP-MS	ACOG
Wang et al. (47)	China	CC	NS	427	NS	427	3.10 ± 1.26	2.94 ± 1.09	ug/dL	ICP-MS	(69)
Wu et al. (17)	China	RC	NS	59	NS	2,115	4.33 ± 1.94	3.74 ± 1.11	ug/dL	AAS	ACOG
Yazbeck et al. (48)	France	PC	NS	106	NS	865	2.20 ± 1.40	1.90 ± 1.20	ug/dL	AAS	(48)

AAS, atomic absorption spectrophotometer; CC, case-control; CS, cross-sectional; ICP-MS, inductively coupled plasma mass spectrometry; ICP-OES, inductively coupled plasma optical emission spectrometry; KSA, Saudi Arabia; NC, nested cohort; NCC, nested case-control; NICE, National Institution of Care Excellence; NS, not stated; PC, prospective cohort; PIXE, proton-induced X-ray emission; RBC, red blood cell; RC, retrospective cohort.

### 3.3. Results of systematic review

There were 13 cohort studies, eight case-control studies, and four cross-sectional studies. All 25 studies were further assessed *via* the Newcastle-Ottawa Scale (NOS) quality assessment, of which four cross-sectional studies were evaluated with modified NOS. Overall, 19 reports were rated high quality, and six were rated moderate. Detailed scores can be accessed in Supplementary Tables 1.1–1.3.

### 3.4. Results of meta-analysis

**Figure 2:**
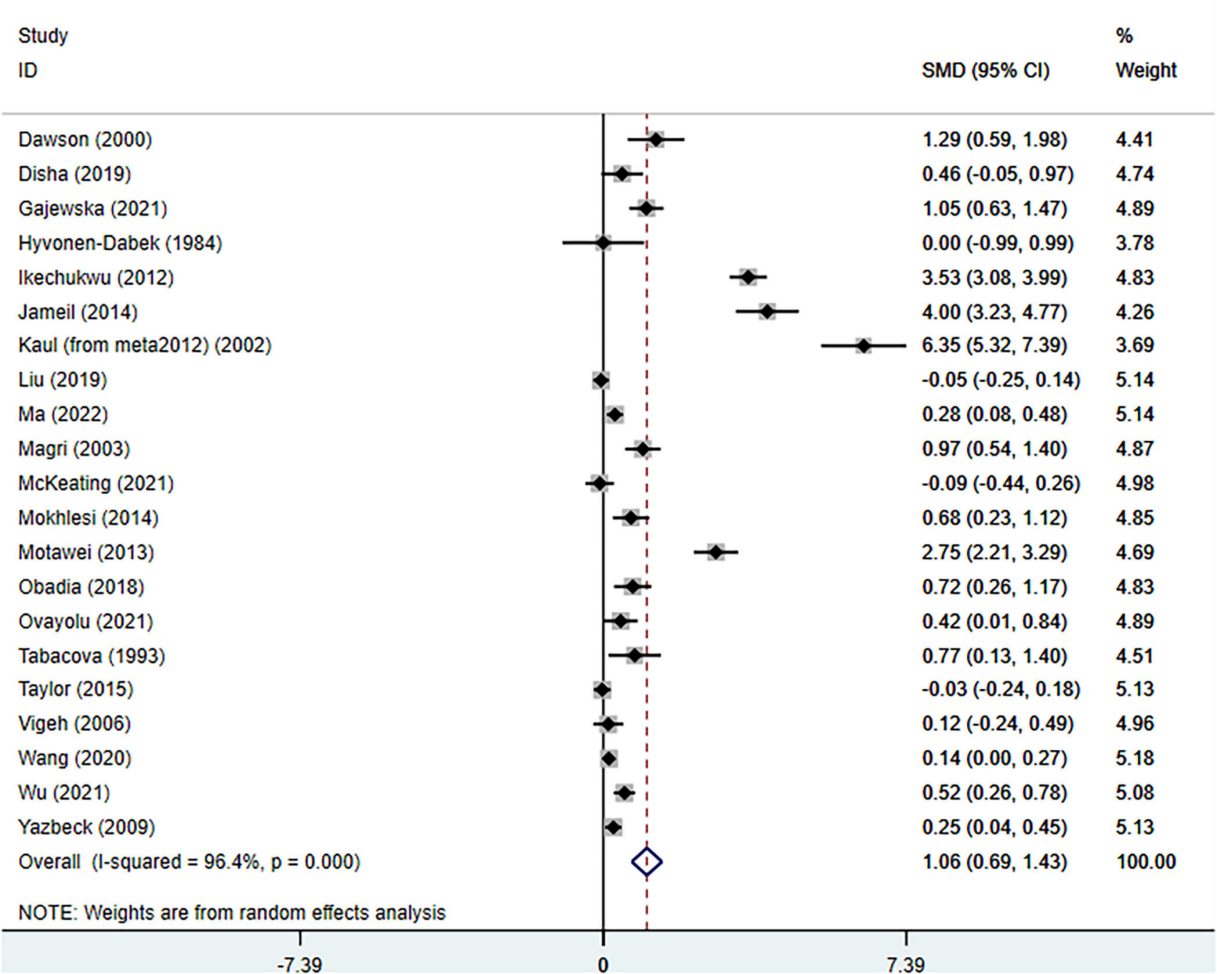
The forest plot of correlation between maternal lead exposure levels in preeclamptic and healthy pregnant women.

**Figure 3:**
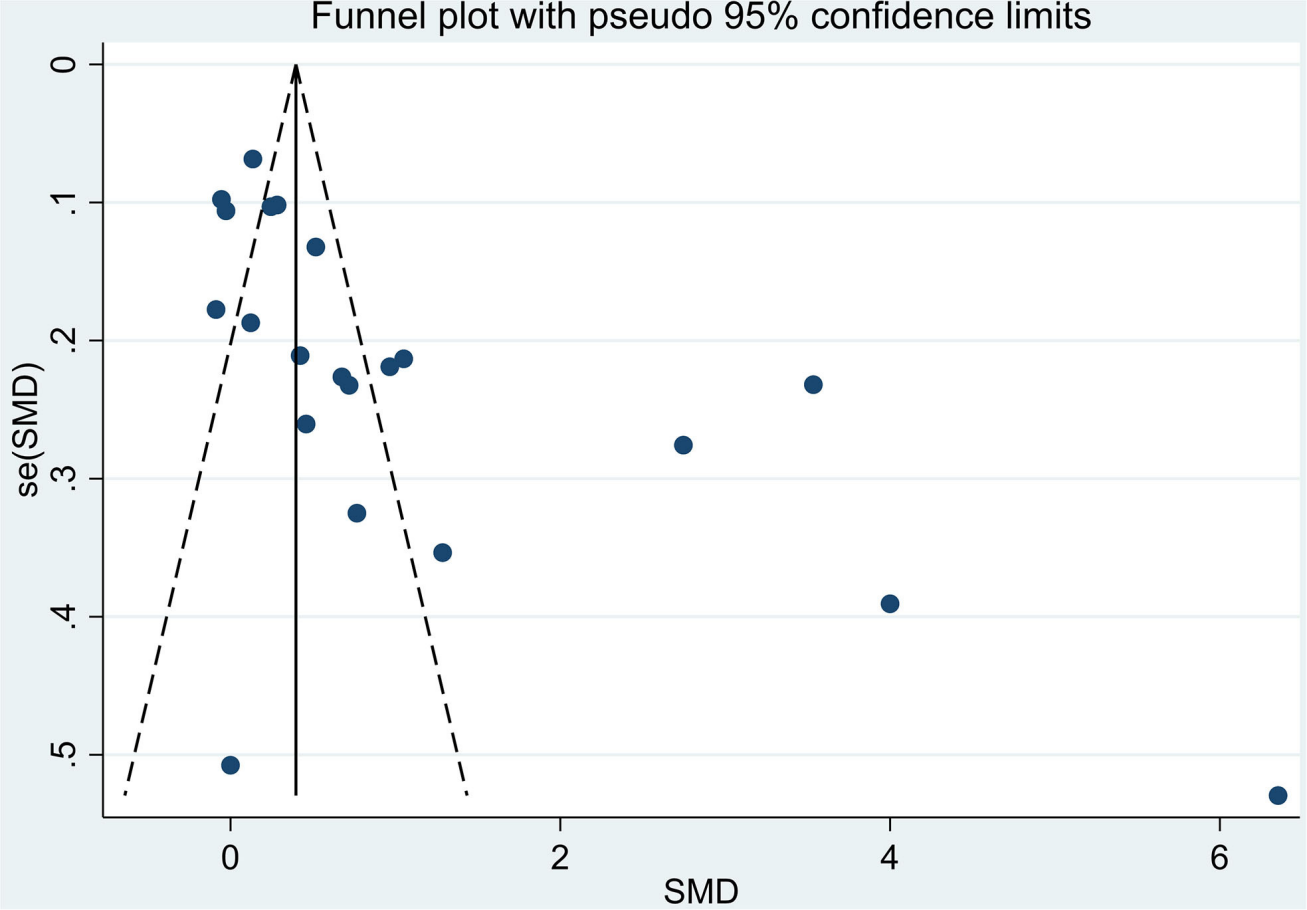
The funnel plot to assess publication bias.

The total number of participants involved in the meta-analysis was 12,531 from 21 reports. The number of healthy pregnant controls was much more compared to the case group. Preeclamptic women accounted for 1,533, while non-preeclamptic pregnant women were more than 7-fold more (1,533 vs. 10,998). The single largest study with 3,976 participants was extracted from a prospective birth cohort in the US in 2015 (16). The overall result showed that maternal lead exposure in preeclamptic women was significantly higher than that of healthy pregnant control (SMD: 1.06, 95% CI 0.69, 1.43); (*I*^2^ = 96.4%; *P* = 0.000), see Figure 2. The funnel plot indicated significant publication bias which can be seen in Figure 3. Begg's test and Egger's test were applied to quantitatively assess the publication bias (*z* = 3.47, *p* = 0.001; *t* = 3.87, *p* = 0.001; see Supplementary Figure 1, Supplementary Table 2). The leave-one-out sensitivity analysis (Supplementary Figure 2) showed that Wang et al. and Ikechukwu et al. reports reversely contributed to the pooled result (37, 47).

### 3.5. Results of meta-regression

Meta-regression was performed as a result of significant heterogeneity between studies. Different geographical locations, measurement methods, types of study design, and blood samples of lead were further tested for potential causes of heterogeneity. However, the results showed that none of them was the major contributor (*P*-value of location: 0.07; *P*-value of different measurements: 0.37; *P*-value of study types: 0.65; *P*-value of sample types: 0.73). Detailed information can be seen in Supplementary Figure 3.

### 3.6. Results of sub-group analysis from a geographic perspective

Despite having evaluated the effect of geographical backgrounds on the general result, we further made a sub-group analysis to see how different the results were in different regions and continents. European studies made up the largest part of the whole study, with 5,325 participants (318 preeclamptic women vs. 5,007 healthy pregnant control). By contrast, African studies only contributed a minor part (428 participants evenly divided), but the effect size was disproportionally large (SMD 2.32; 95% CI 0.60, 4.05); (*I*^2^ = 97.00%; *P* = 0.000). The Asian studies were the most recent and had the lowest heterogeneity with *I*^2^ = 60%; *P* = 0.06; (SMD 0.30; 95% CI 0.12, 0.49). The fifth group consisted of studies from USA and Australia, and there was a significant between-study heterogeneity (SMD 1.74; 95% CI 0.25, 3.24; *I*^2^ = 98.00%; *P* = 0.000). More information is available in Supplementary Figure 4.

We further applied the cut-off value of maternal blood lead level at 5 μg/dL as the Center for Disease Control and Prevention (CDC) of the USA has recommended as the safe range (49). Eleven studies with both the case and control groups whose mean values lower than 5 μg/dL were included for further analysis (14, 16, 17, 20–22, 36, 40, 44, 47, 48). The pooled result showed that the SMD: 0.25, 95% CI 0.09, 0.40; *I*^2^ = 75.00%, *P* = 0.002. More detailed information can be seen in Supplementary Figure 5.

## 4. Discussion

This systematic review and meta-analysis primarily focused on whether there is an association between maternal lead exposure and preeclampsia. The combined results have demonstrated that higher blood lead levels (BLL) are associated with preeclampsia (PE). This is generally consistent with some existing research (25, 50). Lead exposure is associated to adverse maternal and fetal outcomes. The Center for Disease Control and Prevention (CDC) of the USA recommended the safe range of BLL in pregnant women to be 5 μg/dL or less. Globally, the WHO recommends a safety limit of 10 μg/dL BLL (51). In the view of geographical sub-group analysis, the associative trend is more prominent in Africa. This may be associated to rapid industrialization, environmental pollution, diet differences, lifestyle (*kohl*, the black eye cosmetic containing lead sulfide), and poor community awareness in the last decade. Asian studies had relatively lower heterogeneity as three out of the four studies originated in the same country. Apart from geographical differences, sample type, methods of measurements, study designs may also affect the overall effect and give rise to the heterogeneity. We therefore applied meta-regression, but no variables were identified to cause the between-study heterogeneity.

Mothers with advanced age were more likely to develop a steeper increase in BLL than younger mothers, particularly in the latter half of their pregnancy. This coincides with the onset of preeclampsia (52). In this view, it is plausible that higher lead accumulated in advanced maternal age women, disposing them to developing preeclampsia. Furthermore, as the lead was known to cross the placenta freely during pregnancy, there are studies focusing on identifying the association between increased maternal BLL and fetal outcomes. A higher risk of spontaneous miscarriage in the early trimester, and stillbirth in the mid-/late-trimester were also observed in several studies (53, 54). Lead has been shown to induce hyperglycemia and glucose intolerance in pregnant women, which has also been observed in animal studies (33, 55). An association between maternal lead exposure and very preterm birth was identified in a large cohort. Moreover, early-life environmental exposure to lead is related to neurodevelopmental disorders, asthma, and obesity. Interestingly, gender differences were found in 949 mother-child pairs research. Male neonates were at higher risk of preterm delivery even if maternal lead exposure was low (56, 57). In an Iranian study, a negative correlation between intrauterine lead exposure and neonatal birth weight was reported recently (58). Furthermore, a study from Mexico demonstrated that girls born from mothers with lead exposure during pregnancy may have delayed puberty, poor pubic hair, and breast growth (59). These show the sustained effect of lead on mothers and offspring, particularly female offspring. A prenatal animal study also supported this opinion as they have found that maternal lead exposure may create a non-genetic adaptive mechanism to protect against reproductive impairment. This process involves imprinting or cell programming and can persist for a long time (52). As lead was believed to interfere with iron absorption, maternal lead exposure was investigated for anemia developed during pregnancy. However, there has been insufficient evidence to consolidate the correlation by far (32).

Despite several studies demonstrating an association between heavy metals and risks of developing preeclampsia for decades, recent reviews have not included lead or other heavy metals as potential risk factors for preeclampsia (1–3, 17, 20, 44). One reason is that in most western countries, women are at very low risk of suffering from environmental or occupational lead exposure or other heavy metal exposure. In this sense, we divided studies into several groups based on geographical location, assuming there may be differences between each group due to ethnic, economic, lifestyle differences. These differences may also partly explain the significant between-study heterogeneity in this systematic review and meta-analysis. Another reason is that the raised awareness leads to less use of lead-contained food, water, or cosmetics. Fewer women nowadays are exposed to environmental and/or occupational lead without protection. This makes research more difficult to discover significant changes on human beings, and many recent studies turn to focusing on animal study (55).

ACOG stated there is no association between lead exposure and the development of pregnancy-induced hypertension (PIH) (49, 60). However, based on the result we collected, even a low blood lead level (<5 μg/dL) can lead to adverse pregnancy outcomes, including preeclampsia (22, 36, 40). This was further fortified by our sub-group analysis (Supplementary Figure 5). There are different theories to explain the potentially underlying mechanisms. Firstly, the physiological changes in pregnancy facilitate the mobilization of maternal bone lead, contributing to a higher maternal BLL. This triggers a further release of endothelin, a vasoconstrictor involved in the inflammation process, which plays a key role in the pathogenesis of preeclampsia (12, 61). Secondly, an animal study has suggested that long-term lead-contained drinking water can significantly enhance the plasma levels of adrenaline and noradrenaline. This could induce blood hypertension, which is partly responsible for the pathogenesis of preeclampsia (54). Thirdly, high maternal BLL may lead to local changes in miRNA profiles based on research focusing on the cervix (31). As lead can freely cross the placenta, maternal lead exposure during pregnancy could lead to higher *in-utero* lead levels (56). The high levels of umbilical cord blood lead can further trigger changes in fetal miRNA profiles, making it more susceptible to developing maternal preeclampsia and fetal preterm birth or stillbirths. DNA methylation changes were also observed following maternal lead exposure (34, 62). Interestingly, some adverse fetal outcomes are differently associated with fetal sex. DNA methylation is more prominent in female fetuses, while males are at higher risk of pre-term births (34, 57).

Two systematic reviews and meta-analyses focused on the correlation between maternal lead exposure and preeclampsia (25, 50). Kennedy et al. published in 2012, which included only nine original studies. They claimed to search the database from inception to March 2011, but we have found three more articles that met their acclaimed inclusion criteria (14, 24, 45). Poropat et al. study was very well-written, particularly their discussion part. It has been reported that an increment of 1 μg/dL of blood lead was associated with a 1.6% increase in the likelihood of preeclampsia. However, they share a similar problem of not including all the available studies. They have missed at least five studies collected by Kennedy et al. without explanation, indicating that there might be insufficient search in the research (15, 48, 63–65). Another significant mistake was data accuracy. We have found that the review published in 2018 mishandled the results extracted from Ikechukwu et al. study (25). The SD was 12.8 instead of being mistakenly noted as 24.0, and the number of participants in this study was actually 209 instead of 181 which was written in the systematic review (25, 37). The selection bias and inaccurate data extraction may compromise the reliability of the overall result synthesized in this meta-analysis (25).

Compared to the previous research, we have included the most recent and the largest number of studies reporting maternal lead exposure and preeclampsia. This facilitates detailed analysis from different perspectives. We divided the studies into five sub-groups to see how geographic locations impact the overall result and the heterogeneity between studies. We have included all nine reports since 2018, accounting for the latest trends worldwide (17, 20–22, 36, 40, 43, 44, 47).

However, there are several limits to our study. Firstly, despite the unlimited language requirement during the search, we only extracted one non-English written article (written in Persian) (41). This is a shared problem in the previous meta-analysis (50). Secondly, the size of included reports precludes further analysis. We have tried to investigate the correlation between lead and preeclampsia *via* different perspectives, such as dividing the studies into different geographical groups and applying meta-regression to see whether study design, measurement methods, and blood samples have exerted an effect on the overall result. All methods we tried failed to identify any causal effects, nor to significantly minimize the between-study heterogeneity. Lastly, exposure to heavy metals often occurs in mixtures instead of in single forms (20, 66). However, only nine articles in our studies reported other heavy metals (15, 20, 21, 40, 43–47). With more studies reporting the panel of heavy metals, we would be a step closer to exploring the real-world facts.

## 5. Conclusion

In summary, we have found that maternal lead exposure is associated with PE during pregnancy, even at very low levels. More well-designed large cohort studies in the future are needed further to clarify the role of lead on preeclampsia and pregnancy.

## Data availability statement

The original contributions presented in the study are included in the article/Supplementary material, further inquiries can be directed to the corresponding authors.

## Author contributions

Conceptualization and modification and writing back to reviewers: ZZ and FZ. Methodology, resources, and data curation: QY and CL. Software and investigation: ZZ and QY. Validation, project administration, and supervision: FZ and XC. Formal analysis: ZZ and XC. Writing—original draft preparation and funding acquisition: ZZ. Writing—review and editing: XC. Visualization: CL. All authors have read and agreed to the published version of the manuscript.
