# Supplementary material for: A global perspective of correlation between maternal blood lead levels and risks of preeclampsia: An updated systematic review and meta-analysis
Source: Front Public Health. 2022 Dec 23;10:1072052. doi: 10.3389/fpubh.2022.1072052 (PMC9816335; doi:10.3389/fpubh.2022.1072052)
Supplement: Supplementary file 1 [file Table_1.DOCX]

**Table 1.1 Quality assessment using the Newcastle-Ottawa Scale (NOS) for cohort studies**

| **Included studies** | **Selection** | | | | **Comparability** | **Outcome** | | | **Score**  **(0-9)** |
| --- | --- | --- | --- | --- | --- | --- | --- | --- | --- |
|  |  | ② | ③ | ④ | ⑤ | ⑥ | ⑦ | ⑧ |  |
| Dawson et al (2000)^1^ | ★ | ★ | ★ | ★ | ★☆ | ★ | ★ | ☆ | 7 |
| Hyvonen-Dabek et al (1984)^2^ | ☆ | ★ | ★ | ★ | ★☆ | ★ | ★ | ☆ | 6 |
| Ikechukwu et al (2012)^3^ | ☆ | ★ | ★ | ★ | ★☆ | ★ | ★ | ★ | 7 |
| Liu et al (2019)^4^ | ★ | ★ | ★ | ★ | ★★ | ★ | ★ | ★ | 9 |
| McKeating et al (2021)^5^ | ☆ | ★ | ★ | ★ | ★★ | ★ | ★ | ★ | 8 |
| Mokhlesi et al (2014)^6^ | ☆ | ★ | ★ | ★ | ★☆ | ★ | ☆ | ☆ | 5 |
| Rothenberg et al (2002)^7^ | ☆ | ★ | ★ | ★ | ★☆ | ★ | ★ | ☆ | 6 |
| Sowers et al (2002)^8^ | ★ | ★ | ★ | ★ | ★☆ | ★ | ★ | ☆ | 7 |
| Tabacova et al (1993)^9^ | ★ | ★ | ★ | ★ | ★☆ | ★ | ★ | ☆ | 7 |
| Taylor et al (2015)^10^ | ★ | ★ | ★ | ★ | ★★ | ★ | ★ | ★ | 9 |
| Ugwuja et al (2011)^11^ | ☆ | ★ | ★ | ★ | ★★ | ★ | ☆ | ★ | 7 |
| Wu et al (2021)^12^ | ☆ | ★ | ★ | ★ | ★★ | ★ | ★ | ★ | 8 |
| Yazbeck et al (2009)^13^ | ★ | ★ | ★ | ★ | ★★ | ★ | ★ | ★ | 9 |

Selection (maximum 4 stars): ① Representativeness of the exposed cohort; ② Selection of the non-exposed cohort; ③ Ascertainment of exposure; ④ Demonstration that outcome of interest was not present at the start of study;

Comparability (maximum 2 stars): ⑤ Comparability of cohorts based on the design or analysis;

Outcome (maximum 3 stars): ⑥ Assessment of outcome; ⑦ Was follow-up long enough for outcomes to occur; ⑧ Adequacy of follow up of cohorts

“★” represents requirement fulfilled, and score “1” in this section; “☆” indicates not fulfill the requirement or not stated in the study and hence score “0” in this section

**Table 1.2 Quality assessment using NOS for case-control studies**

| **Included studies** | **Selection** | | | | **Comparability** | **Outcome** | | | **Score**  **(0-9)** |
| --- | --- | --- | --- | --- | --- | --- | --- | --- | --- |
|  |  | ② | ③ | ④ | ⑤ | ⑥ | ⑦ | ⑧ |  |
| Bayat et al (2016)^14^ | ★ | ★ | ☆ | ☆ | ★☆ | ★ | ★ | ☆ | 5 |
| Gajewska et al (2021)^15^ | ★ | ★ | ★ | ★ | ★★ | ★ | ★ | ☆ | 8 |
| Jameil et al (2014)^16^ | ★ | ★ | ★ | ☆ | ★☆ | ★ | ★ | ☆ | 6 |
| Ma et al (2022)^17^ | ★ | ★ | ★ | ☆ | ★★ | ★ | ★ | ★ | 8 |
| Obadia et al (2018)^18^ | ★ | ★ | ☆ | ★ | ★★ | ★ | ★ | ☆ | 7 |
| Ovayolu et al (2021)^19^ | ★ | ★ | ★ | ★ | ★☆ | ★ | ★ | ★ | 8 |
| Vigeh et al (2006)^20^ | ★ | ★ | ★ | ☆ | ★☆ | ★ | ★ | ☆ | 7 |
| Wang et al (2020)^21^ | ★ | ★ | ★ | ★ | ★★ | ★ | ★ | ★ | 9 |

Selection (maximum 4 stars): ① Is the case definition adequate; ② Representative of the cases; ③ Selection of controls; ④ Definition of controls

Comparability (maximum 2 stars): ⑤ Comparability of cases and controls based on the design or analysis

Exposure (maximum 3 stars): ⑥ Ascertainment of exposure; ⑦ Same method of ascertainment for cases and controls; ⑧ Non-response rate

**Table 1.3 Quality assessment using modified NOS for cross-sectional studies**

| **Included studies** | **Selection** | | | | **Comparability** | **Outcome** | | **Score** |
| --- | --- | --- | --- | --- | --- | --- | --- | --- |
|  |  | ② | ③ | ④ | ⑤ | ⑥ | ⑦ | **(0-10)** |
| Disha et al (2019)^22^ | ★ | ★ | ★ | ★★ | ★☆ | ★★ | ☆ | 8 |
| Kaul et al (2002)^23^ | ☆ | ☆ | ★ | ★★ | ★☆ | ★★ | ☆ | 6 |
| Magri et al (2003)^24^ | ★ | ★ | ★ | ★★ | ★★ | ★★ | ★ | 10 |
| Motawei et al (2013)^25^ | ☆ | ★ | ★ | ★★ | ★☆ | ★★ | ★ | 8 |

Modified NOS was proposed by Modesti et al. in 2016 ^26^

Selection (maximum 5 stars) ① Representativeness of the sample; ② Sample size; ③ Non-repondents; ④ Ascertainment of exposure (risk factors, maximum 2 stars);

Comparability (maximum 2 stars): ⑤ Comparability of outcomes based on the design or analysis;

Outcome (maximum 3 stars): ⑥ Assessment of outcome (maximum 2 stars); ⑦ Statistical test

**Table 2 Begg’s and Egger’s tests**

**Reference**

1. Dawson EB, Evans, DR, Kelly, R, Van Hook, JW. Blood cell lead, calcium, and magnesium levels associated with pregnancy-induced hypertension and preeclampsia*. Biol Trace Elem Res*. 2000;74(2):107-116.

2. Hyvonen-Dabek M, Nikkinen-Vilkki, P, Dabek, JT. Selenium and other elements in human maternal and umbilical serum, as determined simultaneously by proton-induced X-ray emission*. Clin Chem*. 1984;30(4):529-533.

3. Ikechukwu IC, Ojareva, OI, Ibhagbemien, AJ, et al. Blood lead, calcium, and phosphorus in women with preeclampsia in Edo State, Nigeria*. Arch Environ Occup Health*. 2012;67(3):163-169.

4. Liu T, Zhang, M, Guallar, E, et al. Trace Minerals, Heavy Metals, and Preeclampsia: Findings from the Boston Birth Cohort*. Journal of American Heart Association*. 2019;8(16).

5. McKeating DR, Fisher, JJ, MacDonald, T, et al. Circulating trace elements for the prediction of preeclampsia and small for gestational age babies*. Metabolomics : Official journal of the Metabolomic Society*. 2021;17(10):90.

6. Mokhlesi S, Moghaddam-Banaem, L, Lamyian, M, Alyianmoghadam, N, Safari, K. Prediction of preeclampsia based on blood lead levels in early pregnancy*. Journal of Shahrekord Uuniversity of Medical Sciences*. 2014;15(6):44-53.

7. Rothenberg SJ, Kondrashov, V, Manalo, M, et al. Increases in hypertension and blood pressure during pregnancy with increased bone lead levels*. Am J Epidemiol*. 2002;156(12):1079-1087.

8. Sowers M, Jannausch, M, Scholl, T, Li, W, Kemp, FW, Bogden, JD. Blood lead concentrations and pregnancy outcomes*. Arch Environ Health*. 2002;57(5):489-495.

9. Tabacova S, Balabaeva, L. Environmental pollutants in relation to complications of pregnancy*. Environ Health Perspect*. 1993;101 Suppl 2:27-31.

10. Taylor CM, Golding, J, Emond, AM. Adverse effects of maternal lead levels on birth outcomes in the ALSPAC study: a prospective birth cohort study*. BJOG*. 2015;122(3):322-328.

11. Ugwuja EI, Ejikeme, B, Obuna, JA. Impacts of elevated prenatal blood lead on trace element status and pregnancy outcomes in occupationally non-exposed women*. The international journal of occupational and environmental medicine*. 2011;2(3):143-156.

12. Wu SZ, Xu, HY, Chen, Y, et al. Association of blood lead levels with preeclampsia: A cohort study in China*. Environ Res*. 2021;195:110822.

13. Yazbeck C, Thiebaugeorges, O, Moreau, T, et al. Maternal blood lead levels and the risk of pregnancy-induced hypertension: the EDEN cohort study*. Environ Health Perspect*. 2009;117(10):1526-1530.

14. Bayat F, Akbari, SA, Dabirioskoei, A, Nasiri, M, Mellati, A. The Relationship Between Blood Lead Level and Preeclampsia*. Electron Physician*. 2016;8(12):3450-3455.

15. Gajewska K, Laskowska, M, Almeida, A, Pinto, E, Skorzynska-Dziduszko, K, Blazewicz, A. Lead Levels in Non-Occupationally Exposed Women with Preeclampsia*. Molecules*. 2021;26(10).

16. Jameil NA. Maternal serum lead levels and risk of preeclampsia in pregnant women: a cohort study in a maternity hospital, Riyadh, Saudi Arabia*. International journal of clinical and experimental pathology*. 2014;7(6):3182-3189.

17. Ma J, Zhang, H, Zheng, T, et al. Exposure to metal mixtures and hypertensive disorders of pregnancy: A nested case-control study in China*. Environmental pollution (Barking, Essex : 1987)*. 2022;306:119439.

18. Musa Obadia P, Kayembe-Kitenge, T, Haufroid, V, Banza Lubaba Nkulu, C, Nemery, B. Preeclampsia and blood lead (and other metals) in Lubumbashi, DR Congo*. Environ Res*. 2018;167:468-471.

19. Ovayolu A, Turksoy, VA, Gun, I, Karaman, E, Dogan, I, Turgut, A. Analyses of maternal plasma cadmium, lead, and vanadium levels in the diagnosis and severity of late-onset preeclampsia: a prospective and comparative study*. The journal of maternal-fetal & neonatal medicine : the official journal of the European Association of Perinatal Medicine, the Federation of Asia and Oceania Perinatal Societies, the International Society of Perinatal Obstet*. 2021:1-8.

20. Vigeh M, Yokoyama, K, Ramezanzadeh, F, et al. Lead and other trace metals in preeclampsia: a case-control study in Tehran, Iran*. Environ Res*. 2006;100(2):268-275.

21. Wang Y, Wang, K, Han, T, et al. Exposure to multiple metals and prevalence for preeclampsia in Taiyuan, China*. Environ Int*. 2020;145.

22. Disha, Sharma, S, Goyal, M, Kumar, PK, Ghosh, R, Sharma, P. Association of raised blood lead levels in pregnant women with preeclampsia: A study at tertiary centre*. Taiwan J Obstet Gynecol*. 2019;58(1):60-63.

23. Kaul PP, Srivastava, R, Srivastava, SP, Kamboj, M, Chand, S. Relationships of maternal blood lead and disorders of pregnancy to neonatal birthweight*. Vet Hum Toxicol*. 2002;44(6):321-323.

24. Magri J, Sammut, M, Savona-Ventura, C. Lead and other metals in gestational hypertension*. Int J Gynaecol Obstet*. 2003;83(1):29-36.

25. Motawei SM, Attalla, SM, Gouda, HE, El-Harouny, MA, El-Mansoury, AM. Lead level in pregnant women suffering from pre-eclampsia in Dakahlia, Egypt*. The international journal of occupational and environmental medicine*. 2013;4(1):36-44.

26. Modesti PA, Reboldi, G, Cappuccio, FP, et al. Panethnic Differences in Blood Pressure in Europe: A Systematic Review and Meta-Analysis*. PloS one*. 2016;11(1):e0147601.
