# Supplementary material for: A global perspective of correlation between maternal blood lead levels and risks of preeclampsia: An updated systematic review and meta-analysis
Source: Front Public Health. 2022 Dec 23;10:1072052. doi: 10.3389/fpubh.2022.1072052 (PMC9816335; doi:10.3389/fpubh.2022.1072052)
Supplement: Supplementary file 3 [file Data_Sheet_1.DOCX]

**(a)**

**(b)**

**Supplementary Figure 1.** Publication assessment (a) Begg’s test; (b) Egger’s test.


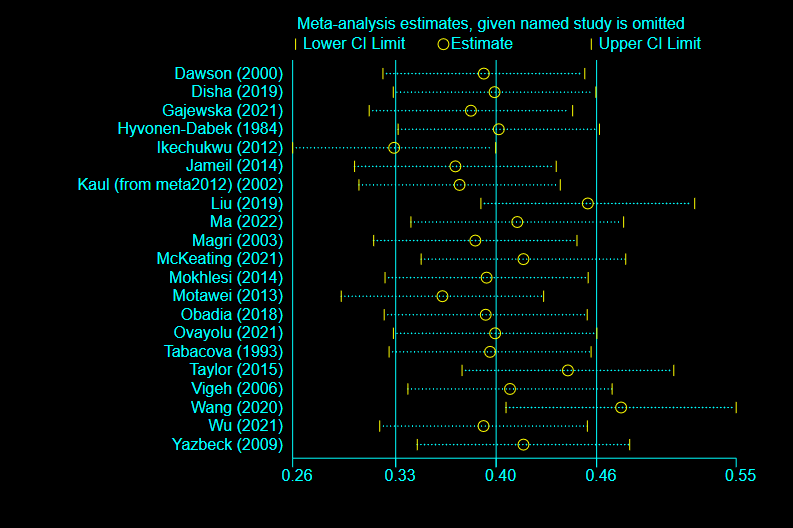


**Supplementary Figure 2.** The leave-one-out sensitivity analysis of included studies

**(a)**

**
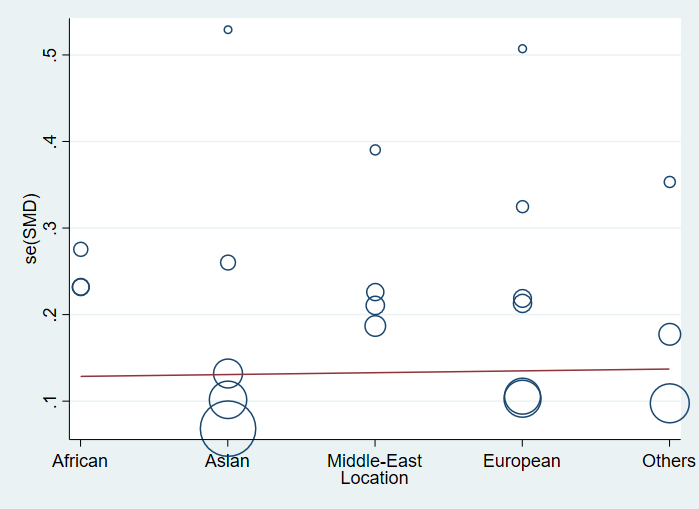
**

**(b)**

**(c)**

**
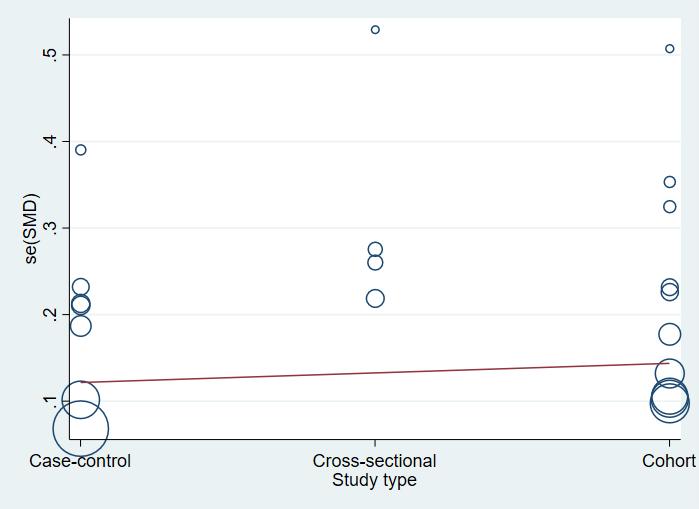
**

**(d)**

**Supplementary Figure 3.** Meta-regression of the included studies: (a). the geographical location of the study (Others stand for studies from Australia, and USA); (b). the measuring methods of serum Pb (AAS, ICP-MS, other methods); (c). the study types (case-control, cross-sectional, and cohort studies); (d). the sample types (blood, plasma, serum, and red blood cell).


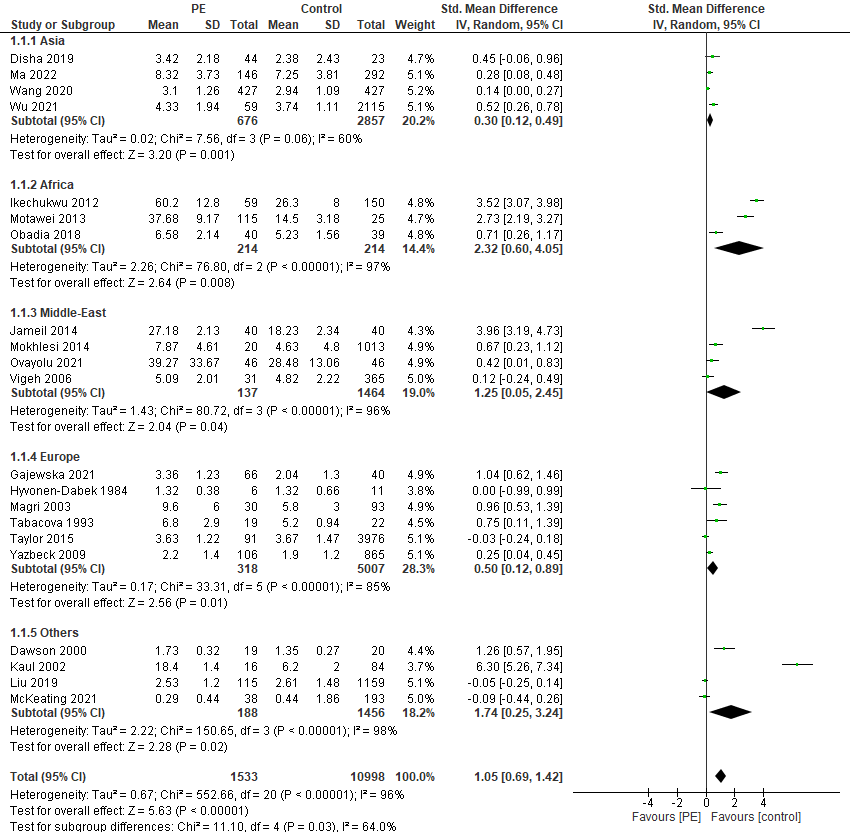


**Supplementary Figure 4.** Maternal blood lead levels in preeclamptic women and healthy pregnant women from Asia, Africa, Middle-East, Europe, USA, and Australia.


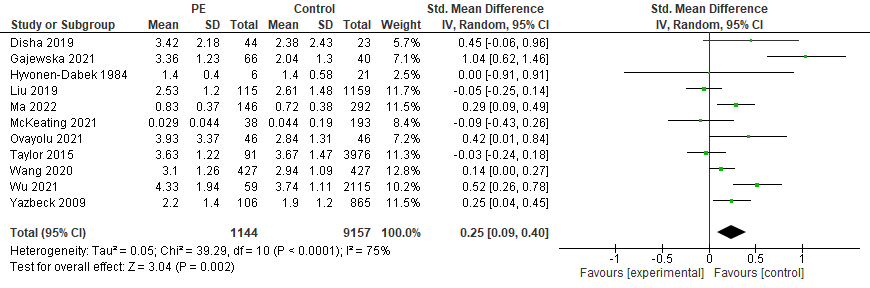


**Supplementary Figure 5.** Maternal blood lead levels in low blood lead levels sub-group.
