# Supplementary material for: A global perspective of correlation between maternal blood lead levels and risks of preeclampsia: An updated systematic review and meta-analysis
Source: Front Public Health. 2022 Dec 23;10:1072052. doi: 10.3389/fpubh.2022.1072052 (PMC9816335; doi:10.3389/fpubh.2022.1072052)
Supplement: Supplementary file 4 [file Data_Sheet_2.DOCX]

**PubMed**

#1 (‘’Lead’’[Mesh]) OR (Lead[title/abstract]) OR (Pb[title/abstract]) OR Lead OR Pb OR (blood Lead)

#2 ("Hypertension, Pregnancy-Induced"[Mesh] OR (pregnancy (toxemia OR toxaemia)) OR preeclampsia OR pre-eclampsia OR eclampsia OR PIH OR (Pregnancy-Induced Hypertension) OR (gestational hypertension))

#3 #1 AND #2

Filters: Humans, Female

N=1491

**Embase**

#1 'preeclampsia'/exp OR preeclampsia

#2 preeclampsia:ti,ab,kw OR 'hypertensive disorder of pregnancy':ti,ab,kw OR 'hypertensive disorder during pregnancy':ti,ab,kw OR 'gestational hypertension':ti,ab,kw OR 'gestational hypertensive disorder':ti,ab,kw OR 'pregnancy induced hypertension':ti,ab,kw OR 'preeclamptic toxaemia':ti,ab,kw OR ‘preeclamptic toxemia’:ti,ab,kw

#3 #1 OR #2

#4 'Lead'/exp AND [female]/lim AND [humans]/lim

#5 Pb:ti,ab,kw

#6 #4 OR #5

#7 #3 AND #6

N=86

**Web of Science**

#1 TS=(Preeclampsia OR pre-eclampsia OR Eclampsia OR HELLP OR hypertensive disorder of pregnancy OR hypertensive disorder complicating pregnancy OR hypertensive disorder during pregnancy OR gestational hypertensive disorder OR HDCP OR gestational hypertension OR pregnancy induced hypertension OR preeclamptic toxemia OR preeclamptic toxaemia OR pre-eclamptic toxemia OR pre-eclamptic toxaemia)

#2 TI=(Lead OR Pb)

#3 #1 AND #2

N=224
